# Simultaneous expression of COX-2 and mPGES-1 in mouse gastrointestinal hamartomas

**DOI:** 10.1038/sj.bjc.6601584

**Published:** 2004-02-03

**Authors:** H Takeda, H Miyoshi, Y Tamai, M Oshima, M M Taketo

**Affiliations:** 1 Department of Pharmacology, Kyoto University Graduate School of Medicine, Kyoto 606-8501, Japan; 2 Banyu Tsukuba Research Institute, Tsukuba 300-2611, Japan

**Keywords:** hamartoma, *LKB1*, *SMAD4*, *CDX2*, COX-2, mPGES-1

## Abstract

Cyclo-oxygenase (COX)-2 is induced in various types of cancer tissues. Here, we demonstrate stromal expression of both COX-2 and microsomal prostaglandin E_2_ synthase (mPGES)-1 in gastrointestinal hamartomas developed in *Lkb1*^+/−^, *Smad4*^+/−^ and *Cdx2*^+/−^mice. These results suggest that PGE_2_ produced by COX-2 and mPGES-1 plays an important role in hamartoma development regardless of the mutated genes causing hamartomas.

Using *Apc*^*Δ716*^ mouse mutant, a model for familial adenomatous polyposis (FAP), we demonstrated earlier that disruption of the gene encoding cyclo-oxygenase (COX)-2 or prostaglandin E_2_ (PGE_2_) receptor EP2 suppresses intestinal polyposis (Oshima *et al*, 1996; Sonoshita *et al*, 2001). These results indicate that PGE_2_ produced through the COX-2 pathway plays an important role in intestinal tumorigenesis. One of the PGE_2_ synthases, microsomal prostaglandin E_2_ synthase (mPGES)-1 appears to be responsible for PGE_2_ production in tumour tissues, because this enzyme is induced and functionally coupled with COX-2 in a human embryonic kidney cell line (Murakami *et al*, 2000). Likewise, COX-2 and mPGES-1 are induced simultaneously in human colorectal cancer tissues (Yoshimatsu *et al*, 2001), intestinal-type gastric adenocarcinomas (Van Rees *et al*, 2003) and *Apc*^*Δ716*^ mouse intestinal adenomas (Takeda *et al*, 2003).

Peutz–Jeghers syndrome (PJS) and juvenile polyposis syndrome (JPS) are autosomal dominant diseases characterised by hamartomatous polyps of the gastrointestinal tract with an increased risk of cancer development. Germ line mutations in the *LKB1* (Hemminki *et al*, 1998; Jenne *et al*, 1998) and *SMAD4* (Howe *et al*, 1998) are responsible for subpopulations of PJS and JPS, respectively. Gene-targeted mice heterozygous for *Lkb1* and *Smad4* develop gastrointestinal hamartomas that have histological characteristics similar to those of PJS and JPS, respectively (Takaku *et al*, 1999; Miyoshi *et al*, 2002). Recently, it has been reported that COX-2 expression is induced in hamartomatous polyps of PJS patients and *Lkb1*^+/−^ mice (Rossi *et al*, 2002; de Leng *et al*, 2003; McGarrity *et al*, 2003), suggesting the roles of COX-2 in hamartoma development. However, COX-2 expression in other types of hamartomas has not yet been examined. Furthermore, it is important to determine whether the expression of mPGES-1 is also induced in hamartoma tissues as in intestinal adenomas and carcinomas.

Here we show that both COX-2 and mPGES-1 are induced in gastric hamartoma tissues of *Lkb1*^+/−^ and *Smad4*^+/−^ mice. In addition, we demonstrate induction of these enzymes also in *Cdx2*^+/−^ mouse colonic hamartomas. These results strongly suggest that production of PGE_2_ is responsible for gastrointestinal hamartoma development as in intestinal adenomatous polyposis.

## MATERIALS AND METHODS

All *in vivo* experiments were carried out with ethical committee approval and met the standards required by the UKCCCR guidelines (Workman *et al*, 1998). Constructions of *Lkb1*^+/−^, *Smad4*^+/−^ and *Cdx2*^+/−^ mutant mice have been described previously (Takaku *et al*, 1999; Tamai *et al*, 1999; Miyoshi *et al*, 2002). We used these mouse models to examine the expression patterns of COX-1, COX-2 and mPGES-1 in hamartomas that were caused by mutations in the putative genes, *Lkb1*, *Smad4* and *Cdx2*, respectively. As the expression of COX-2 and mPGES-1 can be affected by various conditions such as infections, inflammations and host immune responses, it is important to use congenic mice bred in a specific pathogen-free (SPF) facility and compare them with the age-matched littermate controls. The results from these mouse experiments should provide important pieces of evidence applicable to human clinical research. Ages of mutant mice used in this study were 60–66, 76–90 and 20–35 weeks for *Lkb1*^+/−^, *Smad4*^+/−^ and *Cdx2*^+/−^, respectively. Hamartomas were sampled from seven independent mice and used for further analysis.

For immunoblotting, tissue samples were homogenised and sonicated in lysis buffer (50 mM phosphate buffer pH 7.0, 100 mM NaCl, 2 mM EDTA) containing a protease inhibitor cocktail (Roche Diagnostics, Nonnenwald, Penzberg, Germany). After centrifugation at 10 000 × **g** at 4°C for 10 min, 40 *μ*g of the supernatant protein was mixed with 6 × SDS sample buffer (350 mM Tris-HCl, pH 6.8, 36% glycerol, 10% SDS, 600 mM DTT), separated in SDS–polyacrylamide gels and transferred onto PVDF membranes. After blocking with 5% skimmed milk/Tris-buffered saline/Tween 20, membranes were incubated with an antibody for COX-1 (Santa Cruz Biotechnology, Santa Cruz, CA, USA), COX-2, cPGES or mPGES-1 (Cayman Chemical, Ann Arbor, MI, USA) at 1000-fold dilution, or for *β*-actin (Sigma) at 5000-fold dilution. The ECL detection system (Amersham Pharmacia, Uppsala, Sweden) was used to detect the signals.

For immunohistochemistry, tissue samples were fixed in 4% paraformaldehyde, embedded in paraffin wax and sectioned at 4 *μ*m. After pretreatment in 3% H_2_O_2_ in methanol, sections were boiled in 10 mM citrate buffer (pH 6.0) in a microwave oven for 5 min. Sections were blocked with 3% BSA-10% normal serum for 1 h and incubated with the primary antibody for COX-1, COX-2 or mPGES-1 at 400-fold dilution. Immunostaining signals were visualised using Vectastain Elite Kit (Vector Laboratories, Burlingame, CA, USA).

## RESULTS AND DISCUSSION

**Figure 1:**
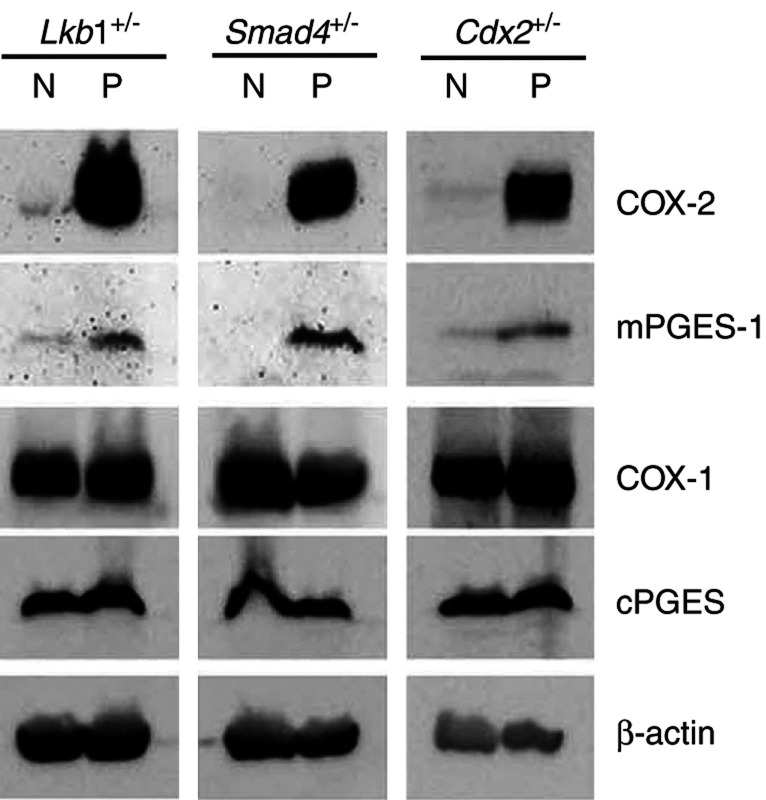
Immunoblotting analysis of COX-1, COX-2, cPGES and mPGES-1 in the normal and polyp tissues. *N*, normal stomach (*Lkb1*^+/−^, *Smad4*^+/−^) or intestine (*Cdx2*^+/−^); *P*, hamartomatous polyp.

Hamartomatous polyp tissues were excised from the stomach of *Lkb1*^+/−^ and *Smad4*^+/−^ mice and from the colon of *Cdx2*^+/−^ mice. We first examined the expression levels of COX enzymes and PGE_2_ synthases in these tumour tissues by immunoblotting (Figure 1

). The expression of COX-2 and mPGES-1 was detected in all hamartomatous polyps, whereas these enzymes were rarely expressed in the normal tissues. On the other hand, COX-1 and cPGES were detected in both normal and hamartoma tissues. These results are consistent with our recent report that COX-1 and cPGES are expressed constitutively in the normal mouse intestines, whereas COX-2 and mPGES-1 are induced in the intestinal polyps of *Apc*^*Δ716*^ mice (Takeda *et al*, 2003).

**Figure 2:**
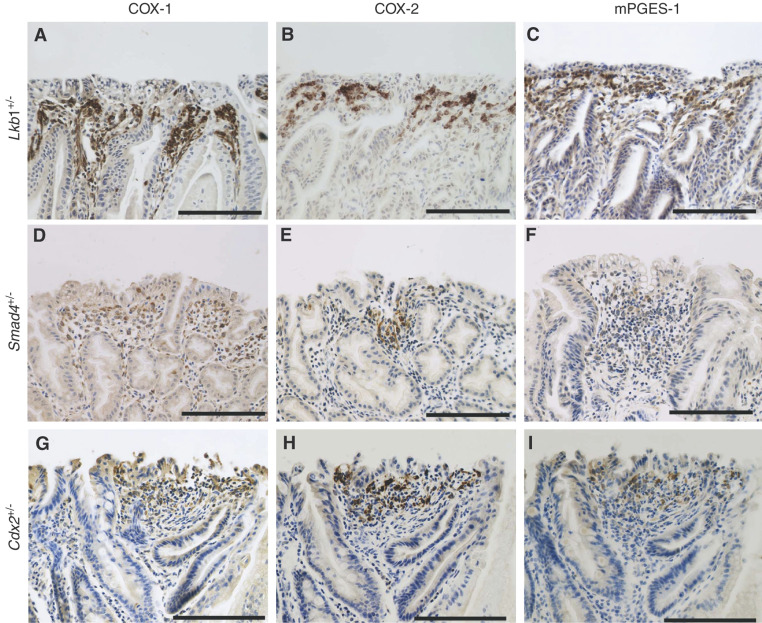
Immunohistochemical analysis of COX-1, COX-2 and mPGES-1 in hamartomas. Expression of COX-1 (**A**, **D**, **G**), COX-2 (**B**, **E**, **H**) and mPGES-1 (**C**, **F**, **I**) in hamartomatous polyps of *Lkb1*^+/−^ (**A**–**C**), *Smad4*^+/−^ (**D**–**F**) and *Cdx2*^+/−^ (**G**–**I**) mice, respectively. Bars; 50 *μ*m.

We next determined by immunohistochemistry the localisation of COX-1, COX-2 and mPGES-1 in the hamartoma tissues of the *Lkb1*^+/−^, *Smad4*^+/−^ and *Cdx2*^+/−^ mice, respectively (Figure 2

). In all samples, COX-1 was expressed in the stromal cells of hamartomas (Figure 2A, D, G) as well as in those of the normal mucosa (data not shown). Expression of COX-2 and mPGES-1 was detected in the stroma of hamartomas near the intestinal lumen, overlapping partly with the COX-1-expressing cells (Figure 2B–I). Moreover, cells expressing COX-2 and mPGES-1 appeared to be the same stromal cells showing a fibroblast-like morphology. There was no apparent difference in the expression patterns of COX-1, COX-2 and mPGES-1 among the hamartomas developing in different mutants. We did not find any difference in the expression levels and cell types among the individual mice of each model. These results indicate that COX-2 and mPGES-1 are induced in the stromal cells, and are consistent with our recent results with the intestinal adenomas in *Apc*^*Δ716*^ mice (Takeda *et al*, 2003). Namely, COX-2 and mPGES-1 are induced simultaneously in the COX-1-expressing stromal fibroblasts. Given that COX-2 and mPGES-1 are functionally coupled (Murakami *et al*, 2000), PGE_2_ levels in the hamartomas should be elevated significantly by simultaneous induction of both enzymes from the basal level that is secured by the COX-1 pathway alone. Although *CDX2* mutations have not been detected in any hereditary hamartoma syndromes, it is conceivable that a subset of sporadic hamartomas contains *CDX2* mutations. Regardless of the mutated genes that caused hamartomas, stromal PGE_2_ production appears to play a key role in the hamartoma expansion.

The PGE_2_ signalling stimulates tumour angiogenesis through the EP2 receptor (Seno *et al*, 2002), increases cell survival and motility (Sheng *et al*, 2001), inhibits host immune responses (Huang *et al*, 1998) and activates epidermal growth factor receptor (EGFR) (Pai *et al*, 2002). Accordingly, it is conceivable that stromal PGE_2_ in the hamartomas contributes to tumour expansion through these effects.

Inhibition of COX-2 by NSAIDs or COX-2-selective inhibitors suppresses intestinal polyposis in *Apc*^*Δ716*^ mice and FAP patients (Oshima *et al*, 1996; Steinbach *et al*, 2000). In addition, administration of COX-2 inhibitor to trefoil factor 1 (TFF1)-deficient mice suppresses gastric adenomas that are caused without Wnt signalling activation (Saukkonen *et al*, 2003). The results suggest that COX-2 induction in the tumour stroma is independent of the molecular mechanism that initiates tumorigenesis in the epithelial cells. These results, taken together, strongly suggest that COX-2 inhibitors, and possibly EP antagonists, are therapeutic agents effective for not only adenomatous polyposis but also hamartomas of the gastrointestinal tract. As hamartomatous polyps can progress into neoplastic tumours (Wang *et al*, 1999), COX-2 inhibitors may also turn out to be cancer chemopreventive agents suitable for hereditary hamartoma syndromes.
